# Pulmonary vein thrombosis and cerebral infarction after video-assisted thoracic surgery of the left upper lobe: a case series

**DOI:** 10.1186/s40981-020-00378-9

**Published:** 2020-09-15

**Authors:** Yosuke Fujii, Yumiko Mori, Kei Kambara, Kiichi Hirota, Masashi Yanada, Shogo Toda, Mitsuko Hashiguchi

**Affiliations:** 1 Department of Anesthesia, Otsu City Hospital, 2-9-9, Motomiya, Otsu, 520-0804 Shiga Japan; 2 Department of Human Stress Response Science, Institute of Biomedical Science, Kansai Medical University, 2-5-1, Shinmachi, Hirakata, 573-1010 Osaka Japan; 3 Department of Thoracic surgery, Otsu City Hospital, 2-9-9, Motomiya, Otsu, 520-0804 Shiga Japan

**Keywords:** Video-assisted thoracic surgery (VATS), Left upper lobe, Pulmonary vein thrombosis (PVT), Thromboembolic complication, Cerebral infarction, Perioperative management

## Abstract

**Background:**

Pulmonary vein thrombosis (PVT) and cerebral infarction are rare but critical complications after video-assisted thoracic surgery (VATS).

**Case presentation:**

We experienced two cases of massive middle cerebral artery infarction after VATS for the left upper lobe. Although the precise source of their embolus was never identified, both cases were clinically suspected PVT. Unfortunately, case 2 died because of progressive cerebral herniation. We decided to perform contrast-enhanced computed tomography routinely after VATS for the left upper lobectomy (VATS-LUL) after these cases. Case 3, a 79-year-old female patient, underwent VATS-LUL for lung cancer. She developed PVT in the stump of the left upper pulmonary vein on postoperative day 4. Anti-coagulation therapy was begun immediately and continued for 3 months. She was free of complications 7 months after the operation.

**Conclusion:**

PVT and cerebral infarction may occur after VATS-LUL. Appropriate postoperative management is required to recognize PVT and to prevent life-threatening stroke.

## Introduction

Cerebral infarction caused by thrombus in the pulmonary vein (PV) stump after lung resection is a rare but lethal complication. According to previous reports, left upper lobectomy (LUL) is a risk factor for thrombus in the PV stump after lung resection [1–3]. In this case series, we describe different outcomes in three patients who developed life-threatening cerebral infarction or pulmonary vein thrombosis (PVT) after video-assisted thoracic surgery (VATS) of the left upper lobe.

## Case presentation

### Case 1

**Fig. 1:**
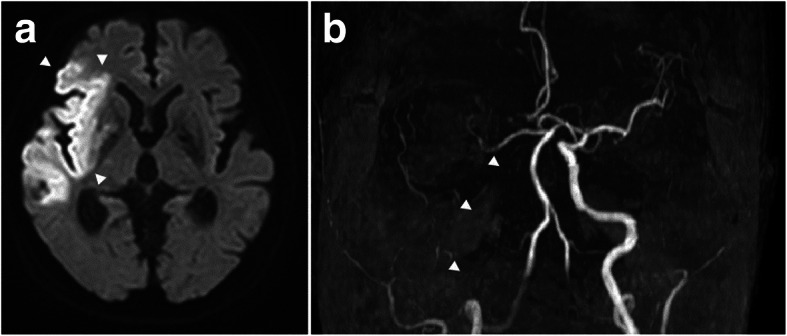
Magnetic resonance imaging (MRI) of case 1. **a** Diffusion weighted MRI revealing edema following a right lobe cerebral infarction (arrowheads). **b** Maximum intensity projection of MRI showing complete occlusion of the right internal carotid artery by a thrombus. Arrowheads indicate the right internal carotid artery with defect

A 77-year-old female patient with hypertension, mild diabetes mellitus, and glaucoma was hospitalized with suspicion of lung cancer in the left upper lobe. She underwent thoracoscopic left upper lobectomy (VATS-LUL). General anesthesia was induced with propofol, remifentanil, and rocuronium and maintained by total intravenous anesthesia with propofol. Intercostal nerve block with 50 mg/20 mL (0.25%) levobupivacaine was performed for postoperative analgesia pain management. The operation time was 172 min. During the operation, 20 mL of blood was lost and 1600 mL (11.3 ml/kg/h) of fluid was infused. There was no perioperative complication including critical hypotension and arrhythmia, and she was discharged directly home on postoperative day (POD) 8. On POD 9, she suffered from sudden onset of left hemiplegia and was admitted to the emergency department. Head computed tomography (CT) revealed right middle cerebral artery (MCA) occlusion and massive cerebral infarction around the Sylvian fissure. Magnetic resonance imaging (MRI) also showed massive MCA stroke and MRI angiography revealed right internal carotid artery (ICA) occlusion (Fig. 1). She was not a candidate for endovascular treatment because she was home alone and symptom onset time was uncertain. She was treated with combination of edaravone and heparin. Electrocardiogram did not show perioperative arrhythmia. Transthoracic echocardiography and plain chest CT could not detect any thrombus. Her left hemiplegia slightly improved but remained.

### Case 2

**Fig. 2:**
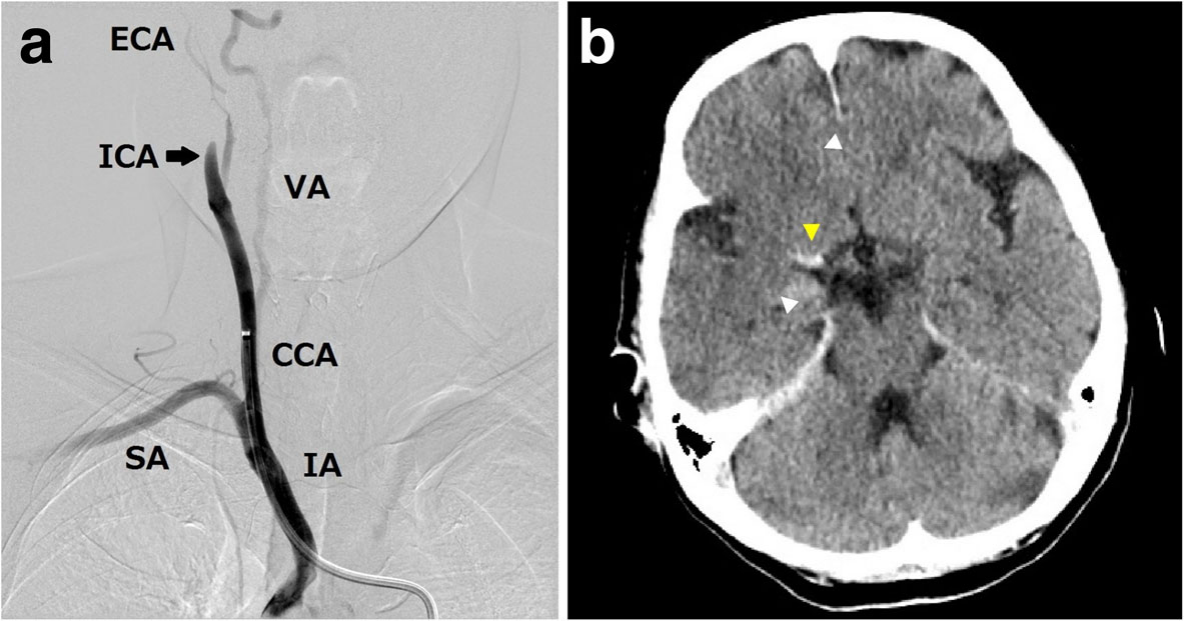
Thrombosis of the right internal carotid artery (ICA) in case 2. **a** Angiogram of the right cerebral circulation showing occlusion of the right ICA (arrow). **b** Head computed tomography after thrombectomy revealing cleared ischemic lesion (white arrowheads) and a thrombus at the right middle cerebral artery (yellow arrowhead). IA, innominate artery; SA, subclavian artery; CCA, common carotid artery; VA, vertebral artery; ICA, internal carotid artery; ECA, external carotid artery

A 71-year-old female patient with dyslipidemia and a history of cured non-tuberculous mycobacterial infection underwent VATS segmentectomy of the left upper division of the left lung for lung cancer. General anesthesia was administered, and intercostal nerve block for postoperative pain relief was performed following protocols the same as those used in case 1. The operation time was 222 min. During the operation, 30 mL of blood was lost, and 1100 mL (6.8 mL/kg/h) of fluid was infused. There was no perioperative complication, and she was free of perioperative hypotension and arrhythmia. She developed sudden weakness in her left leg during walking in the ward and advanced to left hemiplegia on POD 4. Head CT did not detect any obvious sign of stroke, but CT angiography revealed right ICA occlusion. Interventional radiology also showed right ICA occlusion (Fig. 2a), and mechanical thrombectomy was attempted. The following angiography showed recanalization of the right ICA, but more distal vessels (M1 segment of the MCA) was occluded. Head CT after intervention showed massive MCA stroke and a hyperdense middle cerebral artery sign (Fig. 2b). Contrast-enhanced computed tomography (CECT) for chest was proposed to evaluate the source of thrombus, but it was refused by the family. The life-threatening brain edema developed the next day after endovascular intervention and she became unconscious. Life-saving craniotomy was performed on POD 7, but she died due to the progression of intracranial hypertension and cerebral herniation on POD 13.

### Case 3

**Fig. 3:**
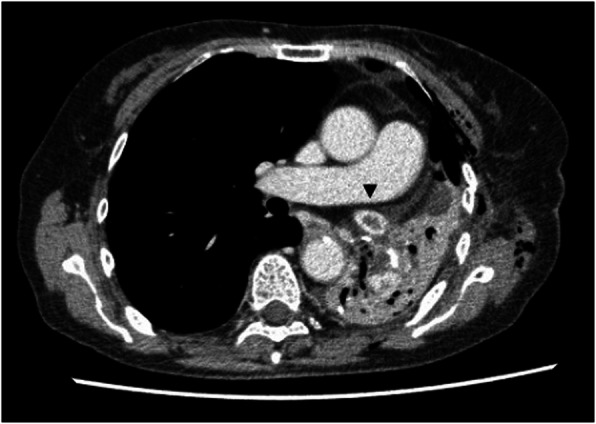
Contrast-enhanced computed tomography showing a thrombus in the left upper pulmonary vein stump in case 3 (arrowhead)

A 79-year-old female patient with hypertension and dyslipidemia was hospitalized with suspicion of the LUL lung cancer. She underwent VATS-LUL. General anesthesia was administered, and intercostal nerve block for postoperative pain relief was performed following protocols the same as those used in cases 1 and 2. The operation time was 282 min. During the operation, 1100 mL of blood was lost because of pulmonary artery injury. The total fluid infusion was 3800 mL (17.4 ml/kg/h); 5 units red blood cell (600 mL) and 1 unit fresh frozen plasma (120 mL) were transfused. There was no sign of perioperative hypotension and arrhythmia. Based on the above two cases, we suspected PVT as the cause of cryptogenic cerebral infarction after VATS of the left upper lobe or division. Therefore, we performed CECT on POD 4, which revealed a thrombus in the stump of the left upper pulmonary vein (Fig. 3). Anti-coagulation therapy with oral warfarin and intravenous heparin was initiated immediately, and no thrombotic event occurred during hospitalization. Three months after the beginning of anti-coagulation therapy, the thrombus in the left PV stump could not be detected on CECT and oral warfarin was discontinued. She was free of any complications 7 months postoperatively.

## Discussion

Perioperative cerebral infarction is a critical complication, and its incidence is dependent on the specific type of surgery performed [4, 5]; incidence of ischemic stroke and the in-hospital mortality rate associated with perioperative stroke after lobectomy or segmentectomy has been reported to be 0.6% and 32.6%, respectively [4]. Since the first case was reported in 1989, several cases of cerebral infarction after VATS-LUL have been reported [6–8]. Recent studies have reported the incidence of PVT and the resulting cerebral infarction after VATS-LUL as 6.7–17.9% [3, 9, 10] and 1.9–4.6% [1, 3], respectively, and revealed that LUL was an independent risk factor of perioperative stroke [1]. Its etiology of PVT has not been completely solved; several mechanisms of thrombus formation, such as the PV stump after VATS-LUL was longer than the other PV stumps and lower bloodstream velocity and more turbulent flow in the dissected PV stump than that in other PV stumps, were suggested [3, 9, 11]. Endothelial injury or surgical staples also can play an important role in thrombogenesis in the PV stump after lung resection [12]. A recent study showed that central vascular ligation of PV stump may reduce intravascular exposure of the staple and contribute to a short PV stump and then contribute to reduce incidence of PVT [10].

Optimal antithrombotic therapy and preventive approaches for PVT remains unestablished, it should be noted that early recognition of PVT and anticoagulant therapy is important to prevent massive cerebral embolism [12]. The induction time and the duration of anti-coagulation therapy are difficult to determine because PVT can occur even after the next day of discontinuation of anticoagulant therapy [13, 14] and can be a possible cause of cerebral embolism anytime after lung resection. In some institutions, they had implemented routine postoperative anti-coagulation therapy in patients following VATS-LUL. Along with those protocols, they changed the perioperative pain management strategy from epidural anesthesia to intercostal nerve block to prevent epidural hematoma in those patients [15].

The incidence of cerebral infarction after VATS-LUL (1.9–4.6%) is similar to the incidence of stroke caused by atrial fibrillation (approximately 5% 2-year age-adjusted incidence) [16]. Lifelong anti-coagulation therapy has been shown to reduce the risk of atrial fibrillation [17]; therefore, it may be a promising option for preventing cerebral infarction after VATS-LUL as long as the risk of bleeding is acceptable.

We report three patients who underwent VATS for left upper lobe and developed thromboembolic complications. The patients in case 1 and case 2 developed life-threatening massive cerebral infarction without obvious cues of PVT. They had no clinical manifestation of perioperative atrial fibrillation, which is one of the major causes of stroke. Clinicians should consider PVT when patients present with cryptogenic stroke or systemic emboli after VATS-LUL [1, 2]. An early definitive diagnosis is critical to rescue the patient and to prevent severe complications. The patient in case 3 had asymptomatic PVT confirmed by CECT. We experienced case 1 and case 2 within a month, which made us change the follow-up strategy to evaluate PVT after VATS-LUL. This helped to detect PVT and prevent the stroke in case 3. Most cases of PVT are detected unexpectedly at postoperative follow-ups [7] while searching for the cause of nonspecific symptoms using CECT [18] or due to a high level of plasma D-dimer [19]. Thus, PVT is overlooked and its incidence is underestimated [8]. Our hospital performed 215 VATS pneumonectomy/lobectomy/segmental lung resection from 2015 to 2019; the number of VATS including left upper lobe was 30 LUL, 13 segmentectomy, and 1 left pneumonectomy. The 5-year incidence rate of cerebral infarction was 4.5% and that of clinically confirmed PVT was 2.3%, respectively. These data revealed that incidence rate of thromboembolic complications after VATS for the left upper lobe in our hospital was equivalent to those of previously reported. Although clinical evidence that shows its efficacy is limited, we currently perform CECT on POD 4 after VATS-LUL to evaluate the presence of PVT because thromboembolic complications would be critical to patients’ prognosis. According to the trends in perioperative major adverse cerebrovascular event associated with non-cardiac surgery, the incidence of ischemic stroke has increased over recent years [20]. LUL is a new risk factor of perioperative stroke, and it is important to diagnose and treat PVT after VATS-LUL.

In conclusion, we reported three cases of postoperative PVT and cerebral infarction after VATS for left upper lobe. The diagnosis and treatment of PVT after VATS-LUL remain challenging. An evidence-based guideline for the detection and management of PVT after VATS-LUL is urgently needed.

## Data Availability

Data relevant to this case series are not available for public access because of patient privacy concerns but are available from the corresponding author on reasonable request.

## Abbreviations

PV: Pulmonary vein
PVT: Pulmonary vein thrombosis
VATS: Video-assisted thoracic surgery
LUL: Left upper lobectomy
CT: Computed tomography
CECT: Contrast-enhanced computed tomography
MCA: Middle cerebral artery
MRI: Magnetic resonance imaging
IA: Innominate artery
SA: Subclavian artery
CCA: Common carotid artery
VA: Vertebral artery
ICA: Internal carotid artery
ECA: External carotid artery

## References

[1] Xie N, Meng X, Wu C (2019). Both left upper lobectomy and left pneumonectomy are risk factors for postoperative stroke. Sci Rep.

[2] Chaaya G, Vishnubhotla P (2017). Pulmonary vein thrombosis: a recent systematic review. Cureus..

[3] Ohtaka K, Hida Y, Kaga K (2013). Thrombosis in the pulmonary vein stump after left upper lobectomy as a possible cause of cerebral infarction. Ann Thorac Surg.

[4] Bateman BT, Schumacher HC, Wang S, Shaefi S, Berman MF (2009). Perioperative acute ischemic stroke in noncardiac and nonvascular surgery: incidence, risk factors, and outcomes. Anesthesiology..

[5] Mashour GA, Shanks AM, Kheterpal S (2011). Perioperative stroke and associated mortality after noncardiac, nonneurologic surgery. Anesthesiology.

[6] Seki M, Endo M, Kidani M, Kobayashi H, Sato H, Noto T (1989). A rare case of left atrial thrombus after left upper pulmonary lobectomy. Nihon Kyobu Geka Gakkai Zasshi.

[7] Ohtaka K, Hida Y, Kaga K (2012). Pulmonary vein thrombosis after video-assisted thoracoscopic left upper lobectomy. J Thoracic Cardiovasc Surgery.

[8] Ikeda H, Yamana N, Murata Y, Saiki M (2015). Thrombus removal by acute-phase endovascular reperfusion therapy to treat cerebral embolism caused by thrombus in the pulmonary vein stump after left upper pulmonary lobectomy: case report. NMC Case Rep J.

[9] Ohtaka K, Hida Y, Kaga K (2014). Left upper lobectomy can be a risk factor for thrombosis in the pulmonary vein stump. J Cardiothorac Surg.

[10] Mizukami Y, Tada M, Adachi H (2020). Cerebral infarction after left upper lung lobectomy with central vascular ligation. J Thorac Dis.

[11] Ohtaka K, Takahashi Y, Uemura S (2014). Blood stasis may cause thrombosis in the left superior pulmonary vein stump after left upper lobectomy. J Cardiothorac Surg.

[12] Hashimoto H, Usui G, Tsugeno Y, et al. Cerebral thromboembolism after lobectomy for lung cancer: pathological diagnosis and mechanism of thrombus formation. Cancers (Basel). 2019;11(4). 10.3390/cancers11040488. 10.3390/cancers11040488 PMC6521235 30959839

[13] Koga T, Mori T, Shibata H, Ikeda K, Shiraishi K, Suzuki M (2016). Two cases of organ infarction due to thrombus in pulmonary vein stump after left lung lobectomy. Jpn J Chest Surg.

[14] Nakano T, Inaba M, Kaneda H (2017). Recurrent cerebral attack caused by thrombosis in the pulmonary vein stump in a patient with left upper lobectomy on anticoagulant therapy: case report and literature review. Surg Case Rep.

[15] Kitajima A, Otsuka Y, Lefor AK, Sanui M (2019). Acute cerebral infarction in a patient with an epidural catheter after left upper lobectomy: a case report. BMC Anesthesiol.

[16] Wolf PA, Abbott RD, Kannel WB (1991). Atrial fibrillation as an independent risk factor for stroke: the Framingham study. Stroke..

[17] Trusz-Gluza M, Filipecki A, Urbańczyk-Świć D (2015). Patients with atrial fibrillation and low risk of stroke: do they really need anticoagulation?. Pol Arch Med Wewn.

[18] Nagaoka E, Yano M, Sugano T, Miyamoto T (2008). Thrombus in the left superior pulmonary vein after left upper pulmonary lobectomy. J Thorac Cardiovasc Surg.

[19] Kamori T, Toyokawa G, Okamoto T (2017). Pulmonary vein stump thrombosis after left pneumonectomy, diagnosed based on a high plasma D-dimer level: a case report. J Thorac Dis.

[20] Smilowitz NR, Gupta N, Ramakrishna H, Guo Y, Berger JS, Bangalore S (2017). Perioperative major adverse cardiovascular and cerebrovascular events associated with noncardiac surgery. JAMA Cardiol.

